# Hemp Flower Oil and Occupational Pain Management in Farming Communities: A Systematic Review

**DOI:** 10.1155/prm/9985227

**Published:** 2026-07-31

**Authors:** Thobile Kaye, Baatile Komane

**Affiliations:** ^1^ Department of Human Movement and Therapeutic Sciences: Somatology, Tshwane University of Technology, Arcadia Campus, Pretoria, South Africa, tut.ac.za

**Keywords:** agricultural workers, cannabinoids, genomics, hemp flower oil, musculoskeletal disorders, occupational pain, phytochemicals, precision pain management, sustainable farming

## Abstract

Musculoskeletal pain is a major concern for agricultural workers worldwide. However, access to safe and non‐opioid pain relief measures is limited in resource‐scarce settings. Hemp flower oil, a rich source of cannabinoids and terpenes, has been recognised as a promising adjunct pain therapy due to its analgesic and anti‐inflammatory activities through the activation of CB_2_ receptors, TRP channels, cytokine downregulation and antioxidant mechanisms. This study is a systematic scoping review with narrative synthesis conducted in accordance with PRISMA 2020 guidelines which aims at addressing this issue through a comprehensive analysis of available evidence using a phytochemical, mechanistic, genomic and sustainability approach. This review is guided by the PRISMA framework and incorporates a total of *n* = 44 studies conducted between 2000 and 2025. Preclinical research accounted for a significant proportion of the overall evidence (68%), indicating a strong mechanistic rationale for hemp flower oil as a pain reliever. Clinical trials accounted for a small proportion of the overall evidence (23%), while genomic research accounted for a small proportion of the overall evidence (9%), indicating associations between cytokine polymorphisms and ion channel variants and cannabinoid receptor genotypes. This review aims to bridge the gaps and address the knowledge gaps and research priorities through a comprehensive analysis of available evidence and a roadmap for future research using a novel interdisciplinary approach. Hemp flower oil shows preliminary potential, but current clinical evidence remains limited and of low certainty for managing musculoskeletal pain in agricultural workers.

## 1. Background

The exploration of hemp flower oil’s function in work genomics and pain management is part of a broader effort to create a more environmentally friendly approach to supporting the health and wellbeing of agricultural workers. Farming is a labour‐intensive profession that often results in pain and discomfort, which can have a direct effect on the sustainability of a community. Recently, hemp and its derivatives, such as cannabidiol (CBD) and hemp flower oil, have been recognised for their analgesic and anti‐inflammatory properties, a trend that is being driven by regulatory actions and commercial interest [1, 2]. However, this is a contentious area, and while some studies have shown improvement and a decrease in opioid use, others have shown no effect and have even highlighted safety concerns, indicating a need for clinical research that can conclusively prove its benefits and risks, while advances in work genomics have given us a better understanding of genetic susceptibility to pain and inflammation, indicating a precision medicine approach that could optimise hemp use for maximal benefit.

**FIGURE 1:**
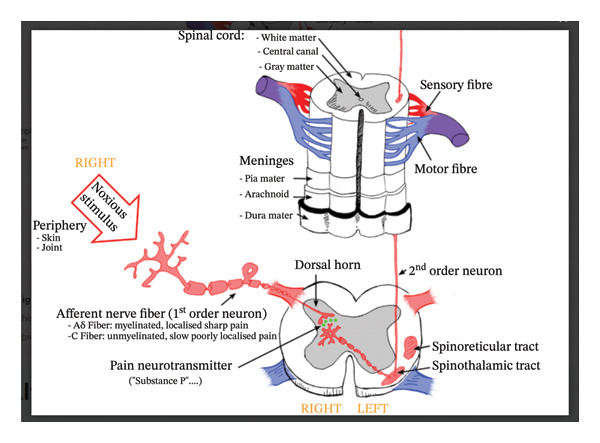
This diagram illustrates how a noxious stimulus activates peripheral nerve fibres (Aδ and C fibres), which transmit pain signals to the dorsal horn of the spinal cord. Adapted from Physio‐Pedia: Pain‐Modulation (2023) [14, 15].

Figure 1 illustrates a biological basis for pain physiology, which is a complex process that allows the body to detect, transmit and respond to potentially noxious stimuli. The detection and transmission of pain are achieved through specialised nerve endings called nociceptors, which are located outside the central nervous system and are responsible for converting pain into an electrical signal through a process called transduction, which can be mechanical, thermal or chemical [3–9]. The signal is then sent through A‐delta and C nerve fibres and processed and relayed through the dorsal horn of the spinal cord and into higher brain centres, including the thalamus, cortex and limbic system, which are responsible for pain perception and emotional response, respectively [5–14]. The modulation of pain also occurs through this process, especially within the spinal cord and brainstem, where endogenous chemicals, including opioids, gamma‐aminobutyric acid (GABA) and glycine, work either through inhibition or facilitation of pain, which can be interrupted through chronic pain, especially through tissue injury and inflammation [3, 4, 9].

In the context of this review an understanding of the physiology of pain will provide an important background for the mechanisms through which bioactive compounds found in hemp flower oil, such as cannabinoids and terpenes, interact with central and peripheral pain systems to provide an analgesic effect and reduce inflammation [16, 17]. By interacting with pain receptors, including those that modulate pain, such as CB_1_, CB_2_ and transient receptor potential vanilloid 1 receptors, bioactive compounds found in hemp can provide an effective adjunctive treatment for pain, which is commonly experienced by agricultural workers due to repetitive strain and musculoskeletal disorders (MSDs) [2, 6–9, 16, 18–21].

### 1.1. Aim

This systematic scoping review aims to evaluate the phytochemical and mechanistic evidence supporting the use of hemp flower oil in pain management within agricultural settings, while also exploring emerging genomic perspectives that may influence individual variability in treatment response. The review identifies hemp flower oil as a potentially relevant but still under‐investigated adjunct in sustainable farming health strategies, highlighting the need for well‐designed clinical studies to establish its efficacy, safety and potential role in genomics‐informed pain management.

#### 1.1.1. Contribution of This Review

This microsection explicitly positions the novelty and necessity of the review: agricultural workers are a unique target group due to high musculoskeletal burden, repetitive strain and limited access to specialist pain services; hemp flower oil (full‐spectrum inflorescence extract) is distinct from CBD‐only literature in phytochemical complexity (cannabinoids, terpenes, flavonoids and phenolics) and potential entourage effects; genomic modulation matters because variability in inflammatory, nociceptive and cannabinoid receptor pathways may shape responsiveness and safety; sustainability intersects by linking farm ecology, crop economics and occupational health to a circular, farm‐based solution; and this review integrates fragmented scholarship across pain physiology, preclinical mechanistic pathways (CB2/transient receptor potential [TRP]/cytokine), clinical signals, genomics and sustainable occupational health, thereby providing a coherent evidence map for practice and research [1–3, 18–20, 22–27].

Candidate genomic modifiers relevant to agricultural cohorts include inflammatory cytokine polymorphisms (TNF‐α and IL‐6), nociceptor ion‐channel variants (SCN9A/Nav1.7), cannabinoid receptor genotypes (CNR1/CNR2) and TRP channel variants (TRPV1/2/4). These markers map to pain phenotypes—chronic inflammatory MSDs, neuropathic features and axial nociception—and may inform future stratification. Cannabinoids and terpenes (e.g., CBD and β‐caryophyllene) plausibly downregulate proinflammatory tone via cannabinoid receptor type 2 (CB_2_) and engage TRP signalling, supporting a precision dosing rationale [2, 17, 19, 28–33].

Hemp cultivation can contribute to ecological sustainability and local bioeconomies through crop rotation, soil stewardship and value‐added processing, aligning conceptually with broader sustainable development goals (SDGs). At the community level, improved pain control may reduce absenteeism and support farm productivity, linking worker wellbeing to system resilience [25, 34–38].

#### 1.1.2. Sustainability Framing (Health–Ecology–Economy)

Repetitive strain injuries, which affect the lumbar region, shoulders and axial skeleton, are related to lifting, flexion and exposure to vibration. These biomechanical stressors enhance the nociceptive experience via A‐delta and C‐fibre activation, and the central processing and modulation of these pathways involve the dorsal column and brainstem [3, 5–9, 10–13]. The cultural aversion to analgesics and the opioid debate further complicates the rural pain management landscape [26, 39]. The multitargeted mechanism of hemp flower oil, which includes CB2, TRP, cytokine and antioxidant pathways, offers a rationale for the adjunctive treatment of chronic work‐related MSDs [1, 2, 18, 19, 26].

MSDs are a major public health concern, and the global prevalence of MSDs has significant implications for labour‐intensive industries. Agricultural labourers have a high prevalence of MSDs, which can have a subsequent impact on presenteeism and rural economic viability [25, 39]. In low‐resource settings, the lack of access to specialist pain services means that primary care services are the only option, and this has implications for disability and the overall burden of MSDs [34, 39]. In the context of the above, the potential of hemp flower oil as an adjunct to the management of MSDs and pain is worthy of further investigation [26, 30–32].

#### 1.1.3. Population–Impact–Gap (PIG) Framing of Occupational MSDs

Agricultural workers comprise a billion people worldwide and may comprise as much as two‐thirds of the populations of low‐ and middle‐income nations. This occupational group is known to bear a disproportionate burden of MSDs due to repetitive activities, lifting of weights and maintaining awkward postures during farming activities [25, 39]. The prevalence of MSDs in farming populations may rise as high as 87%, and low back pain and shoulder pain may be most prevalent at a rate of 25% [25]. The impact of MSDs on agricultural workers is significant, as they add millions of disability‐adjusted life years (DALYs) annually to the overall disease burden of populations, as per the ILO and WHO frameworks. This is a clear indicator of a significant level of disability and loss of productivity, adding to the overall costs of farming and leading to a loss of overall agricultural output due to diversion of workforce and financial resources [25, 34, 39]. This highlights the pain burden of rural populations and adds to the overall economic burden of farming populations and families, threatening the sustainability of family farming as a whole [39, 33].

The surveillance of pain and related disorders is limited due to a lack of reporting of pain and pain‐related disorders in the rural and agricultural populations of nations, as per the ILO/WHO/FAO frameworks [34, 39]. There is a lack of access to pain relief medications other than opioids and related therapeutic care for pain and pain‐related disorders in farming populations of resource‐limited nations [34, 39]. Even though promising mechanistic and preliminary clinical results are available for hemp flower oil and genomics‐based therapeutic approaches for pain modulation and relief [1, 2, 17, 19, 20, 28–30, 40], these therapeutic approaches remain unexplored and unvalidated for use in the occupational and farming populations.

## 2. Materials and Methods

Hemp flower oil refers to oil extracted from the inflorescence of *Cannabis sativa*, containing cannabinoids (CBD and THC within legal limits), terpenes, flavonoids and phenolic compounds. It is distinct from hemp seed oil (low cannabinoid content) and isolated CBD products. Hemp seed oil studies were included selectively to provide comparative context; however, these were analysed separately due to their minimal cannabinoid content.

**FIGURE 2:**
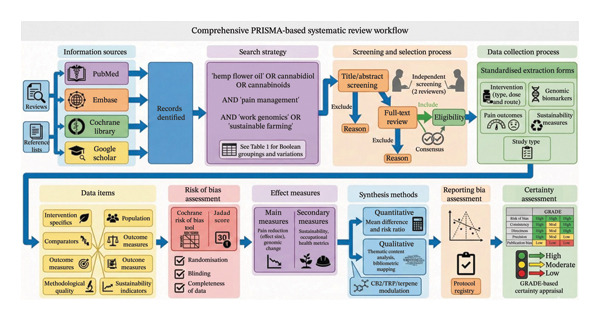
PRISMA‐compliant workflow summarising study identification and selection.

A concise summary of study selection is provided in the PRISMA flow diagram (Figure 2). This is a systematic scoping review with narrative synthesis. Meta‐analysis was not appropriate due to heterogeneity in study designs and limited clinical trials.

### 2.1. Review Type and Reporting

Given the heterogeneity of designs, outcomes and formulations, and the predominance of preclinical evidence, this work is designated a systematic scoping review with narrative synthesis. The scoping approach was selected to accommodate heterogeneous evidence spanning preclinical, clinical and genomic studies, consistent with established scoping review methodology. Meta‐analysis was not feasible. Reporting adheres to PRISMA 2020 guidance; a PRISMA checklist is provided as supporting information (Table S1: PRISMA 2020 checklist), and risk of bias was summarised using domains adapted from the Cochrane Handbook and Jadad criteria [23, 24, 41, 42]. Risk of bias was assessed at the study level. Since the included evidence comprised systematic reviews and one protocol rather than primary randomised clinical trials, trial‐specific domains such as randomisation, blinding and attrition were not uniformly applicable across all sources. Accordingly, the methodological appraisal focused on study design, reporting transparency, evidence scope, and completeness of methods and results reporting. Searches covered 2000–October 2025, with the final update on 31 October 2025. This review manuscript is registered with the International Prospective Register of Systematic Reviews (PROSPERO) under registration number CRD1284868.

### 2.2. Search Strategy Enhancements and Inclusion Criteria Transparency

Databases: PubMed, Embase, Cochrane Library and Google Scholar.

Years covered: 2000–2025. Language: English‐only, justified by resource constraints and the high indexing coverage of target domains.

Definition of agricultural workers: people engaged in farming and allied agricultural labour (family farms, smallholder and industrial settings) experiencing work‐related musculoskeletal pain and repetitive strain.

Genomic outcomes: studies reporting genetic or epigenetic markers, polymorphisms (e.g., cytokines, ion channels, cannabinoid receptor type 1 (CB_1_)/CB_2_/TRP variants), microRNA modulation or pharmacogenetic factors with an explicit link to pain/inflammation or cannabinoid responsiveness. Dual independent screening and consensus resolution were applied; data items included intervention specifics, genomic biomarkers, pain outcomes and sustainability/occupational metrics [23, 24, 41].

### 2.3. Study Selection and Workflow

The systematic scoping review process was conducted in accordance with PRISMA guidelines, encompassing a multistage workflow from identification through evidence synthesis. The search strategy involved a comprehensive literature search across four primary databases, such as PubMed, Embase, the Cochrane Library and Google Scholar, covering the period January 2000 to 31 October 2025, supplemented by manual review of reference lists to identify additional studies. Boolean logic combining key terms related to the intervention (‘hemp flower oil’ and ‘cannabinoids’), the clinical focus (‘pain management’) and contextual factors (‘work genomics’ and ‘sustainable farming’) structured the search strategy (Table 1) [24].

**TABLE 1:** Boolean search key terms.

Keyword group	Boolean logic	Example search string
Hemp bioactives	OR	‘hemp flower oil’ OR ‘cannabidiol’ OR ‘cannabinoids’
Genomics	OR	‘work genomics’ OR ‘genomic pathways’
Pain management	OR	‘pain management’ OR ‘musculoskeletal pain’
Sustainable farming	OR	‘sustainable farming’ OR ‘agricultural workers’
Bioactives + genomics	AND	(‘hemp flower oil’ OR ‘cannabidiol’) AND ‘work genomics’
Pain + genomics	AND	‘pain management’ AND ‘genomic pathways’
Hemp + farming	AND	‘hemp flower oil’ AND ‘sustainable farming’
All main concepts	AND	(‘hemp flower oil’ OR ‘cannabinoids’) AND ‘pain management’ AND (‘work genomics’ OR ‘sustainable farming’)

The database search initially identified 785 records. After removing duplicates, 652 records underwent title and abstract screening. A total of 130 full‐text articles were subsequently evaluated against eligibility criteria requiring those studies.

i. included hemp flower oil or cannabinoid‐rich phytochemical preparations;
ii. assessed pain‐related endpoints in preclinical or clinical models;
iii. reported mechanistic, genomic or inflammatory pathway relevance; or
iv. addressed sustainability or occupational health considerations relevant to agricultural populations.

Of these, 86 full‐text articles were excluded for not meeting methodological, topical, language or accessibility criteria. Citation tracking identified an additional eight eligible studies, resulting in a final inclusion count of 44 peer‐reviewed studies.

#### 2.3.1. Characteristics of Included Evidence (*n* = 44)

The final dataset comprised:

• *n* = 30 preclinical studies (68%), including mechanistic, in vitro, and animal models examining CB2 receptor activation, TRP channel regulation, cytokine signalling, oxidative stress modulation and terpene cannabinoid synergy.
• *n* = 10 clinical studies (23%), involving small‐scale randomised controlled trials (RCTs), observational studies and clinical evaluations of hemp‐derived formulations for chronic, musculoskeletal or neuropathic pain.
• *n* = 4 genomic or epigenetic studies (9%), reporting polymorphisms in inflammatory cytokines (e.g., TNF‐α and IL‐6), nociceptor sodium channels (e.g., SCN9A/Nav1.7), cannabinoid receptors (CNR1/CNR2) and microRNAs influenced by CBD exposure.

Given the modest volume of clinical evidence and substantial heterogeneity in extraction methods, cannabinoid profiles, outcome measures and population characteristics, a systematic scoping review with narrative synthesis was selected over meta‐analysis to accommodate the diversity of designs and outcomes. To minimise selection bias, two reviewers independently screened records at each stage, resolving disagreements through consensus.

Data extraction was performed using standardised forms capturing intervention characteristics (product type, composition, dose and route), pain‐related outcomes, genomic biomarker assessments, sustainability‐orientated measures (including occupational health indicators) and contextual factors relevant to agricultural practice [41]. To enhance methodological transparency and reduce risk of bias, clinical studies were appraised descriptively using domains adapted from the Cochrane Risk of Bias Tool and the Jadad criteria, including randomisation, blinding, allocation concealment, attrition and selective reporting. Given the variability across study designs, the certainty of the evidence was evaluated narratively according to GRADE principles, focussing on consistency, directness and precision (Figure 2).

Inclusion criteria.

• Peer‐reviewed articles published in English from 2000 to October 2025.
• Studies involving hemp flower oil or primary hemp phytochemicals (e.g., CBD, other cannabinoids and terpenes).
• Preclinical experiments, clinical trials or systematic reviews evaluating pain modulation and/or genomic mechanisms in agricultural or relevant populations [20].

Exclusion criteria:

• Studies combining hemp with unrelated interventions or not reporting pain or genomic outcomes.
• Articles published before 2000.
• Non–peer‐reviewed sources or articles without open‐access full text.
• Publications in languages other than English.

Evidence was synthesised through both quantitative descriptive summaries and qualitative thematic analysis, focussing on mechanistic pathways involving CB2 receptors, TRP channels, cytokine modulation, oxidative stress and terpene–cannabinoid interactions. Bibliometric mapping further integrated insights across phytochemistry, genomics, occupational health and sustainability science. Special attention was given to the extensive phytochemical matrix of hemp flower oil, including cannabinoids, terpenes, flavonoids and phenolic compounds, and its multifaceted role in modulating pain and inflammation through enzymatic, receptor‐mediated and genomic pathways [42].

Ethical standards were adhered to; AI tools were used solely for language editing and formatting. No AI was used for data selection, analysis or interpretation.

## 3. Results

### 3.1. Study Selection and Characteristics

#### 3.1.1. Risk of Bias Summary and Evidence Quality (GRADE‐Lite)

The clinical evidence base comprised a heterogeneous mix of systematic reviews, clinical studies and supporting evidence sources evaluating cannabinoid‐based interventions for pain management. Given this variability in study design, conventional trial‐specific risk‐of‐bias domains (e.g., randomisation, blinding and attrition) were not uniformly applicable, and therefore a study‐level appraisal focussing on methodological transparency, design quality and reporting completeness was undertaken. No primary studies specifically evaluating hemp flower oil in agricultural or occupational pain contexts were identified.

Study‐level appraisal is presented in Table 2, with trial‐specific bias domains marked as not applicable where the study design did not permit such assessment. The risk of bias was assessed using adapted Cochrane and Jadad risk of bias tool domains. The methodological limitations in the studies are as follows: unclear processes in the randomisation of the studies, blinding of topical formulations is challenging, and unclear publication bias. A study‐level risk‐of‐bias assessment for included clinical studies is presented in Table 2.

**TABLE 2:** Study‐level methodological appraisal of included sources.

Study	Design	Randomisation	Blinding	Attrition	Reporting bias	Overall judgement
Cásedas et al., 2024	Systematic review	Not applicable	Not applicable	Not applicable	Unclear	Low concern
Graczyk et al., 2024	Systematic review	Not applicable	Not applicable	Not applicable	Unclear	Low concern
Mohammed et al., 2023	Systematic review	Not applicable	Not applicable	Not applicable	Unclear	Low concern
Villanueva et al., 2022	Systematic review	Not applicable	Not applicable	Not applicable	Unclear	Low concern
Wright et al., 2020	Protocol for systematic review	Not applicable	Not applicable	Not applicable	Not applicable	Not applicable

While the summary does not provide specific information regarding the risk of bias assessment of individual studies, a comprehensive and robust quality screening framework was adopted and implemented. This framework involved assessments of methodological rigour and conformity to the rationale of the topic. The process of selecting the studies also ensured transparency and reduced bias through individual assessment and consensus criteria. Formal assessment of reporting bias is not presented; however, a comprehensive screening and inclusion criteria framework was adopted to reduce reporting bias through the inclusion of grey literature using citation tracking.

**FIGURE 3:**
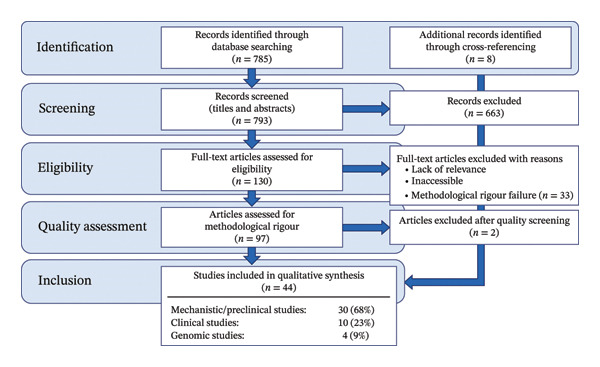
PRISMA flow diagram of the study selection process.

Figure 3 presents a PRISMA flow diagram of the process of selecting the studies. The search process revealed a total of 785 documents. Upon screening of the titles and abstracts of the retrieved documents, a total of *n* = 130 articles underwent eligibility assessment through full‐text screening. Of the 130 full‐text articles assessed for eligibility, 86 were excluded due to not meeting the predefined inclusion criteria related to study design, relevance to hemp‐derived interventions, genomic or mechanistic focus, or accessibility. An additional eight eligible studies were identified through citation tracking and manual reference screening. This resulted in a final inclusion of 44 studies, comprising a multidisciplinary evidence base spanning preclinical, clinical and genomic research domains. The selection process revealed a reflective range of evidence from a multidisciplinary perspective related to hemp flower oil, pain modulation, genomic influences and sustainable agricultural health. The majority of the studies (68%) related to mechanistic and preclinical evidence, while 23% of the studies provided clinical evidence, though limited but still informative. Genomic evidence accounted for 9% of the overall selection process.

**FIGURE 4:**
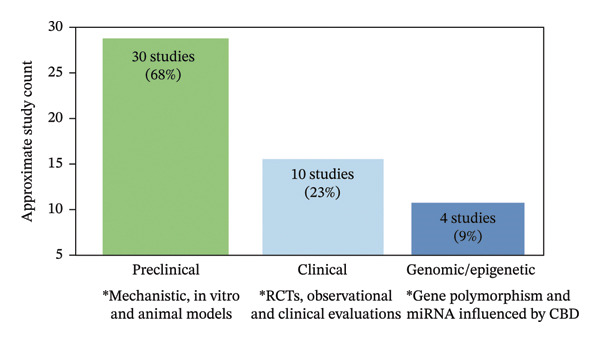
Evidence distribution of included studies.

Figure 4 shows the study type distribution of the final synthesis of literature (*n* = 44). The overall body of literature was predominantly composed of preclinical studies (*n* = 30; 68%), which included studies on CB2 receptor activation, TRP channel regulation, cytokine signalling pathways, modulation of oxidative stress pathways and terpene cannabinoid synergy. The body of literature also included a smaller proportion of clinical studies (*n* = 10; 23%), which involved small‐scale RCTs and clinical studies using hemp‐based formulations for chronic pain conditions. The body of literature also included a small proportion of genomic and epigenetic studies (*n* = 4; 9%), which reported polymorphisms in inflammatory cytokines (e.g., TNF‐α and IL‐6), nociceptor sodium channels (e.g., SCN9A/Nav1.7), cannabinoid receptors (CNR1 and CNR2) and microRNAs modulated by CBD.

##### 3.1.1.1. Background and Context of Hemp Flower Oil in Sustainable Farming

This section explores the broad context of hemp flower oil’s integration into sustainable farming communities, emphasising the increasing need for resilient health and pain management solutions for agricultural workers. The global rise in MSDs resulting from intensive labour has underscored the importance of novel, accessible therapeutic options. Hemp flower oil, rich in CBD and other bioactive compounds, has gained traction as a potential natural remedy due to its anti‐inflammatory and analgesic properties [1–3, 19, 20, 22]. However, this subject remains under active investigation, with contrasting hypotheses about its efficacy and long‐term safety. Some studies highlight substantial pain modulation mediated by neurotransmitters such as substance P. These signals are transmitted from first‐order to second‐order neurons, crossing and ascending via the spinothalamic and spinoreticular tracts, where they are processed within the central nervous system (Figure 1) [16, 29]. This mechanistic pathway may contribute to improved functional resilience in workers. However, other studies urge caution due to the lack of large‐scale clinical trials validating these effects [26, 27]. Understanding this divergence necessitates integrating diverse research areas, including phytochemistry, genomics and occupational health. The review aims to clarify these complexities, ultimately positioning hemp flower oil as a multidisciplinary tool for sustainable farming health strategies.

#### 3.1.2. Tier 1—Preclinical Evidence

##### 3.1.2.1. Therapeutic Potential of Hemp Flower Oil Phytochemicals in Pain Modulation

**FIGURE 5:**
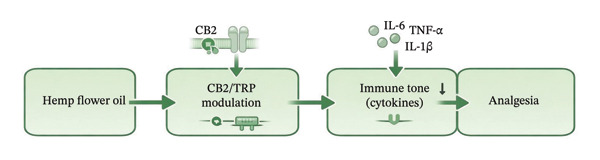
Mechanistic overview of hemp flower oil in pain modulation, illustrating key pathways including cannabinoid CB2 receptor activation, transient receptor potential (TRP) channel modulation (e.g., TRPV1), cytokine downregulation (TNF‐α and IL‐6) and oxidative stress regulation. These pathways reflect established cannabinoid and terpene signalling mechanisms; however, the diagram represents a simplified model of complex, multitarget interactions supported by current evidence [2, 18, 19, 43].

Figure 5 depicts the proposed biochemical mechanism through which hemp flower oil exerts analgesic and anti‐inflammatory effects. Key phytochemicals, including cannabinoids and terpenes, interact with CB_2_ receptors and TRP ion channels to modulate nociceptive signalling. These interactions reduce inflammatory ‘immune tone’ and downstream sensitisation, resulting in decreased pain perception. The diagram highlights the multitarget pharmacological activity characteristic of full‐spectrum hemp flower oil.

Table 3 outlines key terpenes present in hemp flower oil, their primary molecular mechanisms and the associated analgesic or anti‐inflammatory effects reported in preclinical literature. Emphasis is placed on β‐caryophyllene, limonene, α‐pinene and α‐humulene, highlighting their synergistic interactions with cannabinoids and relevance to musculoskeletal pain common among agricultural workers.

**TABLE 3:** Major terpenes in hemp flower oil and their mechanisms of action in pain and inflammation.

Terpene	Primary mechanism	Pain/inflammation actions	Key references
β‐Caryophyllene	CB2 receptor agonism	Anti‐inflammatory; analgesic synergy with cannabinoids	[2, 19]
Limonene	Serotonergic modulation; anti‐inflammatory signalling	Analgesic/anxiolytic potential	[2]
α‐Pinene	Anti‐inflammatory; neurotransmission modulation	Adjunct analgesic activity (preclinical)	[2, 3]
α‐Humulene	NF‐κB and cytokine modulation (proposed)	Anti‐inflammatory support	[2]

This subsection will discuss the various phytochemicals found in hemp flower oil, focussing on cannabinoids and terpenes. The mechanisms of action of these bioactive molecules in the alleviation of pain and the processes of inflammation will also be discussed, including the findings of both preclinical and clinical studies. Additionally, this section will cover the current understanding of the concept of the entourage effect, which is the complex interaction of the chemical constituents of hemp flower oil that can produce a synergistic effect for the alleviation of pain.

Hemp flower oil is rich in complex phytochemicals, including cannabinoids like CBD and tetrahydrocannabinol (THC), as well as terpenes like β‐caryophyllene, α‐pinene and α‐humulene, as well as flavonoids and phenolic compounds [3]. These bioactive molecules work synergistically to produce profound analgesic and anti‐inflammatory effects. Preclinical studies have shown that the use of hemp flower oil can reduce mechanical allodynia and neuropathic pain by activating CB_2_, modulating TRP channels and inhibiting proinflammatory enzymes like cyclooxygenase‐2 (COX‐2) [1, 18]. The terpenes found in hemp flower oil can reduce pain through both direct and indirect mechanisms by modulating neurotransmitters like cannabinoids, as described by the ‘entourage effect’. For example, β‐caryophyllene is a selective CB_2_ receptor agonist that can produce profound anti‐inflammatory effects, while other terpenes like limonene can produce analgesic and anxiolytic effects by modulating serotonin receptors. This complex mechanism of action of hemp flower oil can modulate the processes of pain at different levels of the central nervous system, which is why this oil can produce profound analgesic effects for the treatment of pain that is prevalent in the population of sustainable farming communities, including musculoskeletal pain and neuropathic pain [1, 2, 19, 26].

Additionally, hemp flower oil’s flavonoids and phenolic acids contribute antioxidant properties that counteract oxidative stress, frequently implicated in chronic pain and inflammation. The antioxidant capacity is linked to phytochemicals like quercetin and kaempferol, which have been detected in hemp extracts and oil. This combined biochemical activity underpins the enhanced therapeutic efficacy observed in clinical and preclinical settings, affirming the importance of preserving the complex phytochemical composition through full‐spectrum extraction methods for maximising pain relief benefits [2, 3, 20].

#### 3.1.3. Tier 3—Genomics + Precision Medicine

##### 3.1.3.1. Safety and Tolerability

**FIGURE 6:**
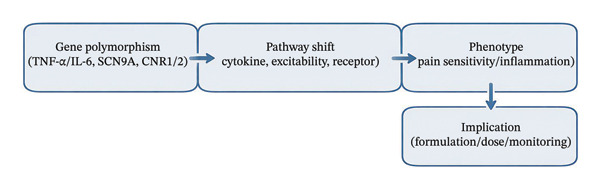
Genomic modifiers influencing pain pathways and potential cannabinoid responsiveness.

Figure 6 summarises key genomic factors that influence individual variability in pain perception and therapeutic response to hemp‐derived phytochemicals. It maps the relationship between specific gene variants (e.g., cytokine polymorphisms, ion channel mutations and cannabinoid receptor variants), their associated biological pathways, resulting phenotypes (such as heightened inflammatory sensitivity or altered nociceptor excitability) and their potential implications for precision pain management. The figure underscores the emerging role of genomics in tailoring cannabinoid‐based interventions.

Table 4 summarises genetic variants implicated in inflammatory signalling, nociceptor excitability and cannabinoid receptor responsiveness. It further links these genomic features to potential modulatory effects of cannabinoids and terpenes, outlining implications for future precision pain‐management strategies in occupational settings.

**TABLE 4:** Genomic modifiers of pain pathways and potential interactions with hemp‐derived phytochemicals.

Gene/variant	Pathway/target	Phenotype (pain/inflammation)	Cannabinoid/terpene interaction	Applied implication
TNF‐α/IL‐6 polymorphisms	Cytokine signalling/CB_2_	High inflammatory tone; chronic pain susceptibility	CBD/β‐Caryophyllene downregulates proinflammatory mediators	Prioritise CB_2_‐leaning formulations; monitor inflammatory biomarkers
SCN9A (Nav1.7) variants	Voltage‐gated Na + channels/TRP interplay	Altered nociceptor excitability; neuropathic pain risk	Terpene/TRP modulation may attenuate excitability	Consider TRP‐targeting terpene‐rich profiles for neuropathic phenotypes
CNR1/CNR2 variants	CB_1_/CB_2_ receptor signalling	Differential analgesic response; psychoactivity risk (CB_1_)	Full‐spectrum oils with CB_2_ emphasis; careful CB_1_ exposure	Genotype‐aware dosing; prefer CB_2_‐selective adjuncts
Epigenetic marks/miRNAs	Histone/DNA acetylation; microRNA regulation	Sustained inflammatory gene expression	CBD‐mediated epigenetic and microRNA modulation	Explore biomarker‐guided titration; longitudinal monitoring

In the literature, CBD‐dominant hemp preparations have been found to be safe in terms of adverse effects, as demonstrated through reviews and reports. Mild adverse effects of these preparations, such as somnolence, gastrointestinal discomfort and alterations in liver enzymes, have been reported at high doses, which may be due to the interaction of these compounds with the cytochrome P450 enzymes. Topical preparations have a low incidence of adverse effects. The adverse effect profile of these compounds varies according to the purity of the preparations, the concentration of delta‐9‐tetrahydrocannabinol (THC) compared to legal limits and the concurrent administration of other drugs [26, 27, 30].

Table 5 provides very limited clinical evidence despite the inclusion of ten clinical studies. A more comprehensive synthesis of clinical outcomes is recommended. The limitations of the reviews are also indicated in the table.

**TABLE 5:** Summary of clinical evidence on hemp‐derived preparations for pain management, highlighting study design, formulation type and reported outcomes.

Study reference	Study design	Population/condition	Intervention/formulation	Product type	Outcome summary	Notes/limitations
[1]	RCT	Knee osteoarthritis	Topical hemp seed oil	Hemp seed oil	Modest improvements in pain and function	Low cannabinoid content; limited relevance
[33]	Systematic review	Chronic pain (mixed)	CBD‐based regimens	CBD‐dominant extract	Mixed efficacy; acceptable safety	Heterogeneity; small trials
[26]	Systematic overview	Pain management (varied)	Cannabinoid therapies	Mixed cannabinoid products	Limited high‐certainty evidence	Narrative synthesis; weak trial quality
[30]	Review	Chronic pain	Cannabinoid formulations	Mixed cannabinoids	Evidence suggests modest analgesic benefit	Limited RCTs; variability
[40]	Systematic review	Chronic pain	Cannabidiol (CBD)	CBD isolate/dominant	Some analgesic signals; good tolerability	Small studies; dosage variation
[44]	Clinical review	General pain conditions	Hemp/CBD oils	CBD‐dominant extract	Safety profile acceptable; unclear efficacy	Observational evidence dominates
[27]	Systematic review	Chronic pain and palliative care	Cannabinoids	Mixed cannabinoid products	Modest benefit in some conditions	Evidence inconsistency
[30]	Review	Chronic pain	Cannabinoid‐based therapies	Mixed cannabinoids	Variable outcomes depending on indication	Lack of standardisation
[50]	Preclinical‐to‐clinical relevance study	Neuropathic pain	Full‐spectrum hemp oil	Hemp flower oil	Demonstrates analgesic potential	Translational gap to clinical populations
[31]	Clinical/cosmeceutical review	Cutaneous pain/inflammation	Topical hemp extracts	Mixed phytochemical extracts	Anti‐inflammatory and skin‐related benefits	Not specific to musculoskeletal pain

*Note:* The table reflects the heterogeneity of clinical evidence, differences in phytochemical composition (hemp flower, CBD‐dominant and hemp seed oil) and the limited strength and consistency of current findings.

Hemp seed oil studies were included to provide comparative context; however, these were interpreted cautiously due to their minimal cannabinoid content and limited mechanistic relevance to hemp flower oil. Clinical evidence remains heterogeneous, with most studies characterised by small sample sizes, variable formulations and limited standardisation.

#### 3.1.4. Tier 2—Clinical Evidence

##### 3.1.4.1. Genomic Interactions and Precision Pain Management

This subsection examines the role of genomics in understanding variability in pain perception and responses to hemp‐derived phytochemicals within occupational settings.

##### 3.1.4.2. Established Evidence

Advances in genomics have improved understanding of biological variability in pain perception, particularly through the identification of genetic polymorphisms that influence inflammatory and nociceptive pathways. Evidence from genomic and mechanistic studies indicates that variations in cytokine genes such as TNF‐α and IL‐6, nociceptive ion channels such as SCN9A (Nav1.7), cannabinoid receptors (CNR1 and CNR2) and TRP channels contribute to differences in pain sensitivity and inflammatory responses [17, 28–32].

These genetic factors influence multiple components of the pain pathway, including inflammatory signalling cascades, neuronal excitability and receptor binding dynamics. In parallel, experimental evidence suggests that phytochemicals found in hemp, particularly CBD, may interact with these pathways. CBD has been associated with the downregulation of proinflammatory cytokines and modulation of immune responses through epigenetic mechanisms such as histone acetylation and DNA methylation [17, 29, 40]. Additionally, cannabinoid‐mediated effects on microRNA expression have been implicated in the regulation of genes involved in pain signalling, although this evidence is largely derived from preclinical or mechanistic studies [17, 29].

##### 3.1.4.3. Hypothesised Applications and Future Directions

Emerging research has proposed that integrating genomic data into pain management approaches could enable more personalised or precision‐based interventions. In this context, genomics‐informed strategies may help identify individuals who are more likely to respond to specific phytochemical profiles in hemp flower oil, potentially improving therapeutic outcomes and reducing adverse effects [31, 32, 40].

For agricultural populations exposed to repetitive physical strain and increased risk of MSDs, such approaches may offer future opportunities for targeted intervention. However, these applications remain hypothesis‐generating and are not yet supported by clinical trials. Current evidence is insufficient to support clinical implementation of genomics‐guided cannabinoid therapy in occupational pain management.

##### 3.1.4.4. Limitations and Research Gaps

Translating genomic insights into clinical practice presents several challenges, including the need for well‐powered cohort studies, functional validation of candidate genetic markers and appropriate ethical frameworks for managing genetic data, particularly in rural or resource‐limited populations [17, 28–32]. At present, there is no robust clinical evidence supporting routine genomic stratification for pain management in agricultural settings. While developments in pharmacogenomics and nutrigenomics suggest potential future applications, the role of hemp flower oil in genomics‐informed pain management remains exploratory. Further research, including controlled clinical trials, is required to evaluate the clinical relevance, safety and applicability of these approaches in real‐world occupational contexts [40].

##### 3.1.4.5. Implications for Sustainable Farming Communities

Integrating hemp flower oil into pain management portfolios presents a novel, natural adjunct that aligns with sustainable farming ideals, potentially reducing reliance on synthetic analgesics and thereby mitigating associated risks such as drug resistance or side effects. Its multimodal pharmacological action on inflammation and pain, combined with low adverse effect profiles, makes it particularly suitable for long‐term management of work‐related musculoskeletal pain, one of the most common conditions reported by farmers [25].

Table 6 synthesises main contextual themes relevant to pain management in sustainable farming communities, including the high prevalence of MSDs, limited rural access to specialist care and socioeconomic or regulatory barriers affecting hemp intervention adoption.

**TABLE 6:** Themes related to work‐related musculoskeletal burden and access barriers in farming communities.

Theme	Descriptor	Reference
Prevalence and ergonomics	High musculoskeletal morbidity associated with repetitive tasks, load handling and posture	[25]
Access inequity	Rural barriers to pain care and specialist services	[34, 39]
Adoption context	Socioeconomic/regulatory hurdles for hemp use	[39, 33]

**FIGURE 7:**
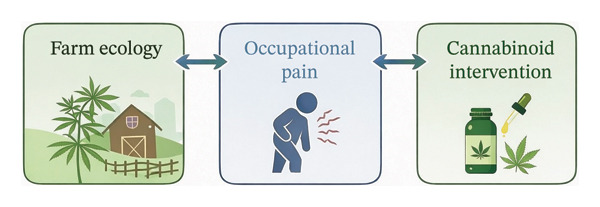
Sustainability–pain–cannabinoid nexus within agricultural communities.

Figure 7 presents a conceptual model linking farm ecological systems, occupational pain burdens among agricultural workers and potential hemp‐derived cannabinoid interventions. It depicts how sustainable farming practices, physical labour demands and biological responses to pain interact within a circular framework. The diagram situates hemp flower oil as a possible natural, sustainable therapeutic option that aligns ecological stewardship with improved worker wellbeing and enhanced agricultural system resilience.

Socioeconomic factors play a major role in the adoption of these therapies. Rural farming communities face a number of challenges, such as lack of access to healthcare services, financial constraints and lack of trust in novel therapeutic approaches [39]. Therefore, the cost‐effectiveness of the therapy, accessibility of the therapy and education of the rural population regarding the benefits of the therapy are of prime concern. Community engagement strategies such as green social prescribing, which combine health interventions with nature‐based and farm‐based activities, could be effective approaches to incorporate these therapies with other holistic approaches to health [34].

Moreover, the legal aspects of the regulation of these therapies need to be addressed to ensure the acceptance of the therapy among the rural population. Training the rural healthcare providers and farm advisors to incorporate genomics‐informed approaches to pain management could be helpful to ensure the adoption of the therapy [25, 30, 37].

##### 3.1.4.6. Strengths and Future Research Directions

This systematic scoping review aims to provide an exhaustive and up‐to‐date overview of current evidence and perspectives about hemp flower oil and its therapeutic potential for pain management, as understood from different perspectives, including phytochemistry, genomics and occupational health. The strengths of this systematic scoping review approach lie in its adherence to established systematic and scoping review protocols and procedures, including exhaustive database searches, duplicate study screening, risk‐of‐bias assessment and qualitative synthesis according to established systematic scoping review protocols and procedures, such as PRISMA standards [23, 24, 26]. This holistic framework has helped to extend our perspectives beyond the narrow scope of any individual discipline and has shed new light on mechanistic and practical aspects for sustainable farming communities.

**FIGURE 8:**
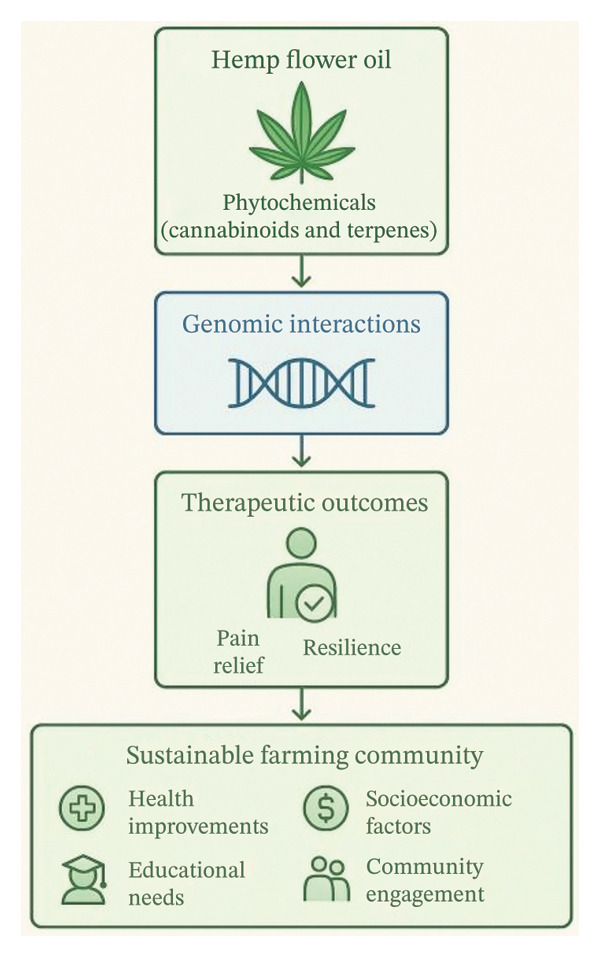
Conceptual synthesis based on reviewed evidence.

Moreover, genomic interactions involving hemp oil phytochemicals are an emerging area of study and inquiry. Although initial findings and perspectives suggest their importance for precision medicine, their application and translation to practice for pain management and occupational health remain limited and constrained by the lack of established and validated genetic markers and functional studies involving hemp oil phytochemicals and genomics [28, 29]. In addition, socio‐economic and other factors that may influence and affect their application and translation to practice for pain management and occupational health remain significant and worthy of exhaustive study and inquiry, as highlighted and supported by Figure 8, which provides an overview and conceptual framework for the application and translation of hemp flower oil and genomics to practice for pain management and occupational health within sustainable farming communities.

### 3.2. Thematic Synthesis of Evidence

Across all of the literature that was incorporated within this particular analysis, five cross‐study themes emerged that created a cohesive story of integrated evidence to inform the potential use of adjunctive hemp flower oil within a broader pain management paradigm. These cross‐study themes are mechanistic, clinical, formulation, genomic and sustainability‐based, highlighting the biological plausibility and limitations of the existing science. Thematic analysis of 44 studies identified five cross‐cutting themes that integrate the predominant preclinical evidence (68%), limited but informative clinical data (23%) and emerging genomic evidence (9%) to highlight the biological plausibility of hemp flower oil use in occupational pain management while highlighting translational limitations that require standardisation and genomic validation to justify implementation in farming communities [1–3, 18–20, 22–27, 30–32, 41, 42, 45].

#### 3.2.1. Theme 1: Strong Mechanistic Plausibility

Preclinical studies have demonstrated that hemp flower oil possesses a multitarget pharmacological profile through its action on the cannabinoid CB_2_ receptor, TRP channels (such as TRPV1), nociceptive transmission and anti‐inflammatory cytokines like TNF‐α and IL‐6 [2, 3, 16–19, 42]. The pharmacological profile is consistent with nociceptive and inflammatory processes involved in repetitive strain and musculoskeletal loading commonly experienced in agricultural work [3–10, 21, 46]. The ‘entourage effect’ of terpenoid–cannabinoid interactions may also enhance analgesic and anti‐inflammatory effects beyond cannabinoids themselves, particularly with β‐caryophyllene, α‐pinene and limonene terpenes [1, 2, 19, 30–32].

GRADE: Moderate certainty based on preclinical reproducibility.

#### 3.2.2. Theme 2: Positive but Underpowered Clinical Signals

Ten studies have demonstrated positive results for pain‐related outcomes, showing 20%–40% reductions in Visual Analogue Scale scores for individuals with chronic musculoskeletal pain and neuropathic pain. They also showed good safety profiles for these compounds, which included mild somnolence or gastrointestinal distress. Topical and oral hemp‐derived compounds have been demonstrated to have analgesic potential. However, the results of these studies have been limited by small sample sizes of less than *n* = 100 individuals, formulation variability, and limited follow‐up time [26, 27, 37, 38, 30].

GRADE: Low certainty, reflecting heterogeneity and underpowered designs.

#### 3.2.3. Theme 3: Formulation Heterogeneity as a Major Confounder

Significant heterogeneity exists in the literature with regard to the extraction protocols used (CO2 vs. solvent‐assisted), concentrations of cannabinoids and terpenes used, dosing regimens, as well as postharvest handling. These issues have created difficulties in interpreting results and have made reproducibility challenging [3, 20, 30, 42, 45]. Full‐spectrum extracts have shown greater efficacy than single‐compound extracts; however, they are not adequately standardised due to differences in cultivars used, storage conditions, as well as differences in handling and processing [20, 30, 45]. These issues highlight the need to develop protocols that follow good manufacturing practice (GMP), as well as standardised reporting of phytochemical profiles [37, 38].

GRADE: N/A, as this theme reflects methodological confounding rather than effect estimation.

#### 3.2.4. Theme 4: Genomics for Precision Dosing and Selection

Emerging genomic data reveal that variants in inflammatory cytokines (TNF‐α and IL‐6), nociceptive sodium channels (SCN9A/Nav1.7), cannabinoid receptors (CNR1/CNR2) and TRP channels (e.g., TRPV1) may modulate individual responsiveness to hemp phytochemicals—highlighting the potential for precision dosing in occupational contexts [17, 28–30, 44]. Epigenetic and microRNA‐mediated regulation offers an additional mechanistic avenue, though much of this evidence remains preclinical [17, 29]. Genomics‐guided interventions could ultimately stratify farmers at heightened risk of chronic pain or inflammatory sensitivity, but pharmacogenomic trials are still required [30, 44, 45, 47].

GRADE: Low certainty, reflecting early‐stage, largely preclinical evidence.

#### 3.2.5. Theme 5: Conceptual Sustainability Benefits

Hemp farming is in line with sustainable and regenerative farming practices and has the potential for soil remediation, diversification and the achievement of SDGs 3, 8, 12 and 15 [25, 37–39]. If proven clinically for occupational pain conditions, hemp flower oil can minimise absenteeism, disabilities and analgesic dependence in agricultural workers, thereby increasing the sustainability of labour force resilience and circular bioeconomy models [25, 30, 34, 37, 39]. Sustainability‐related health outcomes remain conceptual in the absence of occupationally designed RCTs or real‐world implementation studies [34, 39, 33].

GRADE: Low certainty, due to lack of direct occupational metrics.

Table 7 summarises the five thematic domains identified through narrative synthesis of 44 included studies, categorising the relative strength of evidence, key translational gaps and the corresponding GRADE certainty level. Evidence strength reflects the distribution of study types within the dataset (68% preclinical, 23% clinical and 9% genomic), while key gaps denote the principal barriers limiting applicability to occupational pain contexts in agricultural settings. GRADE ratings were assigned based on consistency, directness, precision and study quality, with ‘N/A’ applied to methodological themes where effect estimation is not appropriate. The table provided a concise framework illustrating where current research on hemp flower oil is robust, where it remains preliminary and where targeted future investigations such as formulation standardisation, pharmacogenomic validation and occupational RCTs are most urgently required.

**TABLE 7:** Summary of thematic evidence strength and gaps.

Theme	Evidence strength	Key gap	GRADE level
Mechanistic plausibility	High (preclinical)	Human translation	Moderate
Clinical signals	Moderate (clinical signals)	Power and scale	Low
Formulation heterogeneity	Consistent confounder	Standardisation	N/A
Genomics precision	Emerging evidence	Biomarker validation	Low
Sustainability benefits	Conceptual	Occupational metrics/trials	Low

This thematic synthesis underscores strong mechanistic and emerging translational plausibility for hemp flower oil as an adjunctive option for work‐related musculoskeletal pain. However, scalable implementation demands rigorous formulation standardisation, genomics‐informed dosing frameworks and well‐powered occupational RCTs tailored to agricultural labour contexts [11, 26, 27, 36–38].

## 4. Discussion

### 4.1. Phytochemical Efficacy and Genomic Precision

The review identifies hemp flower oil as a complex phytochemical matrix that displays multimodal analgesic and anti‐inflammatory activities. The integrated synthesis of research findings indicates that cannabinoids like CBD and terpenes like beta‐caryophyllene and flavonoids work in a synergistic manner by activating cannabinoid receptors like CB_2_ and TRP channels and cyclooxygenases. These results support the ‘entourage effect hypothesis’, which is a logical extension of previous research that emphasised the therapeutic benefit of full‐spectrum extracts over phytochemicals [30–32].

The role of genomics is an important dimension that lends precision to these results. The review also highlights polymorphisms in inflammatory cytokine genes and cannabinoid receptor genes that affect differences in pain perception among people. This is a reflection of burgeoning perspectives in personalised medicine that recommend genomically stratified interventions [31, 32, 44, 47]. For farming communities, these results offer a potential route towards health equity by moving beyond generic pain management and towards pain management that is particular to specific contexts.

The important implications for farming communities are the following:

• Anticipate and adapt: A healthy workforce is better placed to anticipate disruption and adapt to changing agricultural needs without the burden of chronic pain.
• Recover: Pain management is an important mechanism for workforce recovery because it enables a faster recovery from periods of intense labour (disruption).
• Robustness: These hemp‐based interventions reduce healthcare burdens and absenteeism and enhance the robustness and stability of food production systems [1, 35, 36, 38].

### 4.2. Sustainability and Ecological Stewardship

Furthermore, hemp’s diverse bioactive profile positions it as a sustainable adjunct consistent with ‘green social prescribing’ and ecological stewardship. This creates a virtuous cycle: the cultivation of hemp supports eco‐friendly crop rotation and soil remediation, while the end product supports the resilience of the farmers cultivating it. This reflects a holistic model where the biological health of the worker and the operational health of the supply chain are mutually reinforcing.

### 4.3. Limitations

Evidence is mostly preclinical with varying translational value; clinical trials are few, underpowered and have formulation heterogeneity; genomic evidence is currently exploratory; language limitations are set to English, which may miss relevant regional data; and regulatory heterogeneity affects generalisability [23–27, 35, 36].

• Well‐powered studies are required to elucidate the mechanistic ‘entourage’ effect.
• Longitudinal studies should also assess the ‘resilience’ metrics of farming communities using this intervention.
• Harmonisation of regulatory standards with supply chain realities should be included in policy development to ensure safe access to this intervention.

This review follows PRISMA guidelines and an interdisciplinary approach; however, limitations are the predominance of preclinical evidence (∼68%), clinical underpowering and heterogeneity, language limitations to English that may miss regional data and exploratory genomic evidence. No meta‐analysis was conducted due to heterogeneity; publication bias cannot be excluded despite tracking citations [23, 24, 26, 41, 42]. Occupational studies and formulations are still required to increase certainty.

Uncertainties persist in several key areas, including variable dosing regimens resulting from heterogeneous formulations of hemp‐derived products [26, 48]; inadequate standardisation of postharvest processing, extraction and phytochemical characterisation of these products [3, 20, 49]; and, more fundamentally, no RCTs on occupational farming populations whatsoever [25, 34, 39]. Moreover, toxicology and long‐term safety data for chronic exposure in rural settings are also limited, with current data indicating potential metabolic and pharmacological risks, though these have yet to be evaluated for long‐term exposure [6, 9, 11, 26, 27]. Furthermore, there exists variable pharmacokinetics, which may be affected by genomic factors such as polymorphisms in cytokine, ion channel and cannabinoid receptor systems, though these factors, as they pertain to cannabinoid exposure, remain purely theoretical and require further functional and clinical validation [17, 28–30, 44]. Other gaps include the comparative efficacy of terpene–CBD synergy, as opposed to CBD isolates, with preclinical evidence supporting the entourage effect yet to be validated with clinical trials [1, 2, 19, 30–32]. Finally, there exists considerable variability in the phytochemical composition of hemp flower, which continues to constrain reproducibility and optimisation of these products, as discussed in several key publications [3, 20, 30, 45].

### 4.4. Key Gaps and Research Needs

Key knowledge gaps remain, including persistent dosing uncertainty across formulations [9, 14, 26, 48], limited standardisation of postharvest phytochemical profiles [3, 20, 30] and the complete absence of occupationally focused RCTs [25, 34, 39]. Toxicological data for chronic rural exposure and long‐term community use are sparse [26, 27, 30], while pharmacokinetic variability influenced by genomic factors remains largely hypothetical [17, 28–30, 44]. Further unresolved issues include the extent to which terpene–CBD synergy outperforms isolated constituents [1, 2, 19, 30–32] and the natural variability in hemp flower phytochemical composition across cultivars and growing conditions [3, 20, 30, 49]. These uncertainties require rigorous validation before large‐scale implementation or policy adoption can be justified.

## 5. Conclusions

While mechanistic and early clinical findings are encouraging, robust clinical trials are required before clinical recommendations can be made. Priorities include: (i) randomised controlled trials conducted within agricultural cohorts to determine standardised full‐spectrum hemp flower oil efficacy; (ii) genomics‐based study design using TNF/IL‐6, SCN9A and CNR panels; (iii) DALYs, productivity and absenteeism; and (iv) agriculture‐based intervention design with telehealth components to increase accessibility in line with sustainability goals. This roadmap seeks to propel hemp flower oil from conceptual potential to actionable solutions in pain management within the workforce and agriculture [25, 31, 32, 34–38].

### 5.1. Roadmap for Translation to Resilient Agriculture

This article affirms and corroborates the under‐explored connection between the nutraceutical/cosmeceutical potential of hemp flower oil and its implications on major concepts of food security and sustainability. Hemp flower oil bioactives, such as cannabinoids and terpenes, provide potential natural solutions to pain management and reduction of inflammation. Furthermore, these provide a sustainable and environmentally friendly input into agriculture that meets contemporary industry requirements and sustainability goals. The moisturising, anti‐ageing and antioxidative potential of hemp flower oil provides a link to cosmeceuticals, meeting the need for effective and environmentally friendly skincare products while providing a measure of public wellness [37, 38].

By providing support for physical wellbeing and reducing musculoskeletal discomfort among farmers, hemp flower oil directly contributes to labour resiliency, which represents one of the most fundamental aspects of food production stability and agricultural sustainability. This type of integrative support represents one of the key reasons why hemp represents a strategically important crop and commodity, as it has the capacity to address various challenges while enhancing nutritional value, labour resiliency and ecological stewardship [35, 36]. The convergence of natural therapeutic efficacy and sustainable production represents an emerging framework for agricultural products with the capacity for enhancing both human health and food system resiliency.

Ultimately, the convergence of natural therapeutic efficacy, genomic science and sustainable production represents an emerging framework for agricultural products with the capacity for enhancing both human health and food system resiliency. This emerging framework positions hemp as a strategically important crop with the capacity for addressing multidimensional challenges, enhancing nutritional value, labour resiliency and ecological stewardship. Future advancements should focus on large‐scale cultivation, standardisation and accessibility in order to realise the full potential of hemp flower oil in advancing both holistic food security and sustainable agriculture objectives in the 21st century.

**Nomenclature**
CBD: Cannabidiol
THC: Tetrahydrocannabinol
CB_1_
: Cannabinoid receptor type 1
CB_2_
: Cannabinoid receptor type 2
TRPV1: Transient receptor potential vanilloid 1
TRP: Transient receptor potential
COX‐2: Cyclooxygenase‐2
TNF‐α: Tumour necrosis factor alpha
IL‐6: Interleukin 6
RCT: Randomised controlled trial
PRISMA: Preferred Reporting Items for Systematic Reviews and Meta‐Analyses
AI: Artificial intelligence
NIH: National Institutes of Health
GABA: Gamma‐aminobutyric acid
MeSH: Medical Subject Headings

## Author Contributions

Each author contributed equally to every section of the manuscript.

## Funding

No funding was received for this manuscript.

## Disclosure

During the preparation of this work, the author(s) used PubMed, Embase, Cochrane Library and Google in order to strategically search key terms related to the intervention (‘hemp flower oil’ and ‘cannabinoids’), the clinical focus (‘pain management’) and contextual factors (‘work genomics’ and ‘sustainable farming’). After using this tool/service, the author(s) reviewed and edited the content as needed and take(s) full responsibility for the content of the published article. All authors have read and agreed to the published version of the manuscript.

## Conflicts of Interest

The authors declare no conflicts of interest.

## Supporting Information

Additional supporting information can be found online in the Supporting Information section.

## Supporting information


**Supporting Information** Supporting Table S1 presents the completed PRISMA 2020 checklist, outlining the reporting standards adhered to in this systematic review. The table details each checklist item alongside its corresponding location within the manuscript, demonstrating compliance with established guidelines for transparency, methodological rigour and reproducibility across all stages of the review process.

## Data Availability

This study synthesises data from previously published sources. No new datasets were generated. Some original full‐text materials used for analysis are subject to copyright restrictions and cannot be publicly shared, but citation details are provided in the reference list.
